# Hemodynamic Evaluation of Residual Cavity Growth in a Basilar-Tip Cerebral Aneurysm Post-coiling Using Silent Magnetic Resonance Angiography (MRA): A Case Report

**DOI:** 10.7759/cureus.75585

**Published:** 2024-12-12

**Authors:** Toru Satoh, Megumi Sasaki, Kana Murakami, Yudai Abe

**Affiliations:** 1 Department of Neurological Surgery, Ryofukai Satoh Neurosurgical Hospital, Fukuyama, Hiroshima, JPN

**Keywords:** aneurysm coiling, fluid dynamics, intracranial aneurysm/therapy, magnetic resonance angiography (mra), remnants

## Abstract

Coil embolization of cerebral aneurysms often encounters challenges in achieving complete filling of the aneurysm sac due to complex shapes and hemodynamic factors, frequently resulting in the formation of a residual cavity (RC) at the aneurysm neck. The hemodynamic mechanisms underlying RC formation and growth, however, remain poorly understood. Computational fluid dynamics (CFD) analysis, combined with silent MRA free from contrast agents and metal artifacts, offers a promising approach to elucidate these mechanisms, potentially enhancing the clinical management of cerebral aneurysms post-coiling. Herein, we report a case of a basilar-tip aneurysm treated with coil embolization, where sequential silent MRA and CFD analysis were employed to investigate hemodynamic factors driving rapid RC growth. Initial RC formation was attributed to coil compaction driven by flow impingement at the aneurysm neck onto the neo-endothelial surface, contributing to vertical growth. In contrast, secondary flows detached from the main inflow jet were observed in distal regions of the RC, leading to flow stagnation, wall vulnerability, and subsequent horizontal expansion of the aneurysmal wall. This case highlights the role of secondary detached flows in RC enlargement, emphasizing their potential to weaken the aneurysm wall and drive sac expansion. CFD analysis using silent MRA is a valuable tool for understanding RC hemodynamics and post-coiling management for cerebral aneurysms.

## Introduction

In the coil embolization of cerebral aneurysms, occlusion is primarily achieved through blood flow stagnation and subsequent thrombus formation within the coiled aneurysm sac [1-3]. However, due to the complex geometry of aneurysms, achieving complete filling of the sac is often challenging. When a residual cavity (RC) at the aneurysm neck enlarges post-coiling, retreatment may become necessary. Hemodynamic factors, such as repetitive flow impingement causing coil compaction at the aneurysm neck, are considered potential contributors to RC enlargement [2-4]. Additionally, factors intrinsic to the aneurysm sac, including degradation of unstable thrombus tissue within the coil mass and increased wall vulnerability, have been reported though the underlying mechanisms remain unresolved [5,6]. RCs formed after coil embolization are not well visualized on time-of-flight magnetic resonance angiography (MRA) due to metal artifacts. In contrast, silent MRA, which does not require contrast materials, minimizes these artifacts, enabling relatively clear visualization of RCs [7,8].

In this study, we conducted a time-course analysis of RC morphology in a basilar-tip aneurysm treated with coils, using silent MRA. To investigate the mechanisms underlying RC formation and growth, computational fluid dynamics (CFD) was applied to analyze the hemodynamic environment within the RC. Our findings demonstrated that RC formation and central growth were primarily driven by flow impingement, which led to coil compaction at the neo-endothelial surface. In contrast, peripheral RC enlargement was associated with increased aneurysm wall vulnerability induced by secondary flows detached from the main inflow jet at the aneurysm neck. To the best of our knowledge, this is the first report to hemodynamically suggest the role of secondary flow detached from the primary jet flow in RC growth following coil embolization of cerebral aneurysms. These findings highlight the potential of CFD analysis to elucidate the hemodynamic mechanisms of RC formation and growth, offering valuable insights to enhance post-coiling management for cerebral aneurysms.

## Case presentation

A woman in her 70s, with an unruptured basilar-tip cerebral aneurysm, was treated by coil embolization. The procedure employed a Target XL-360 coil (16 mm × 50 cm; Stryker Corporation, Kalamazoo, MI, US) to frame the aneurysm, which had expanded to 14.4 × 15.2 × 15.0 mm with a neck measuring 11.4 × 6.47 mm. Thirteen coils were deployed, achieving a volume embolic rate of 23.4%.

**Figure 1:**
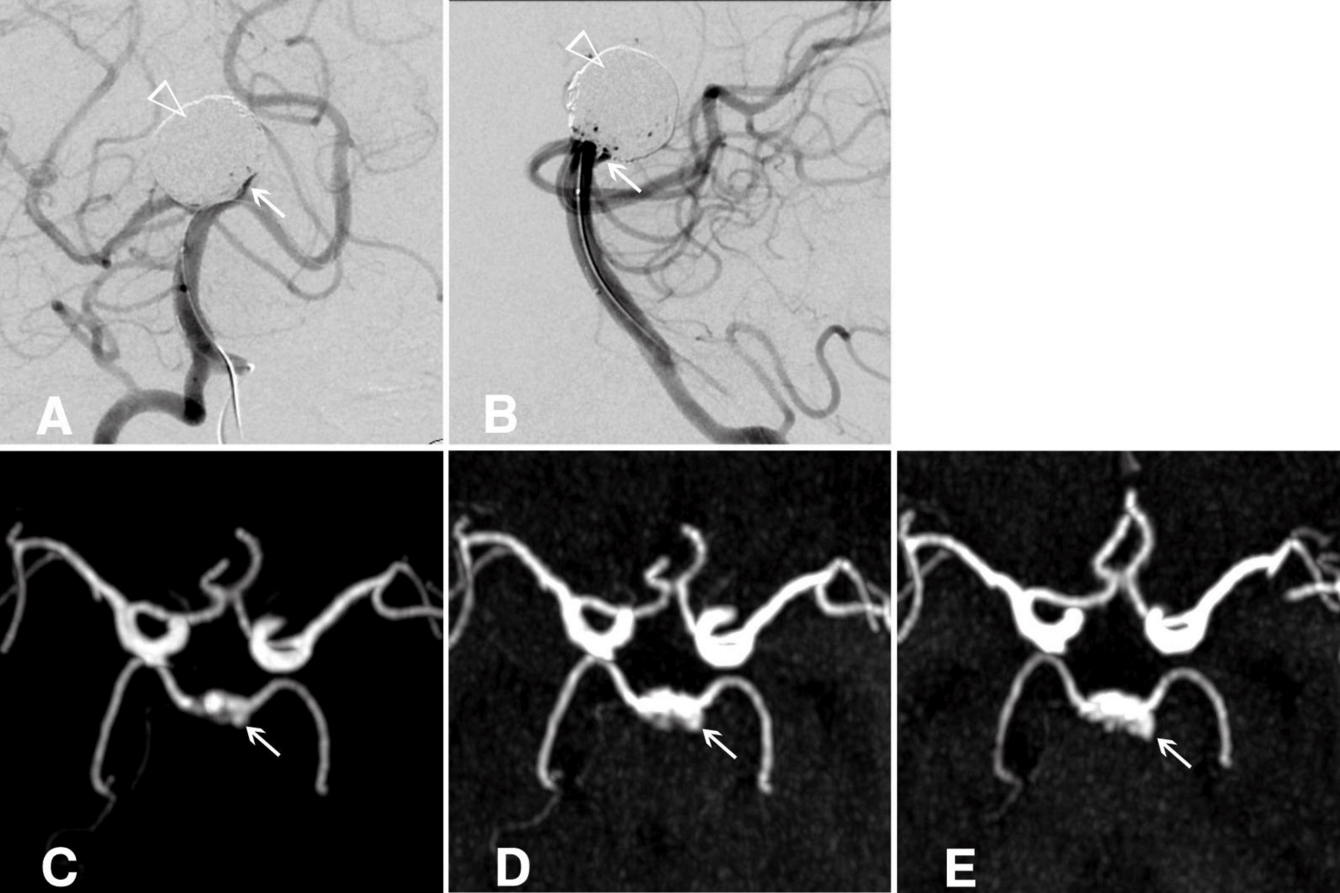
Residual cavity in an unruptured basilar-tip cerebral aneurysm post-coiling A, B) Post-procedural angiograms showing a minimal residual cavity (RC) with no evidence of blood flow stagnation within the aneurysm. The empty white arrowhead marks the coiled dome, and the white arrow highlights the RC. C, D, E) Maximum intensity projection images from sequential silent MRAs demonstrate the progressive formation and growth of the RC at 14 days, 6 months, and 1 year post-treatment, respectively. The white arrow indicates the RC.

Post-procedure, only a minimal residual cavity (RC) was observed, with no significant blood flow stagnation within the aneurysm, indicating a favorable initial outcome (Figures 1A, 1B). Fourteen days after the procedure, silent MRA revealed RC formation, which continued to enlarge over six months and one year of follow-up (Figures 1C-1E). Although retreatment was considered, observation with silent MRA was chosen due to the patient's decreased renal function. The patient remained asymptomatic, with no aneurysm rupture, and passed away from an unrelated condition two years after the procedure. Serial 3D silent MRA images documented RC progression.

**Figure 2:**
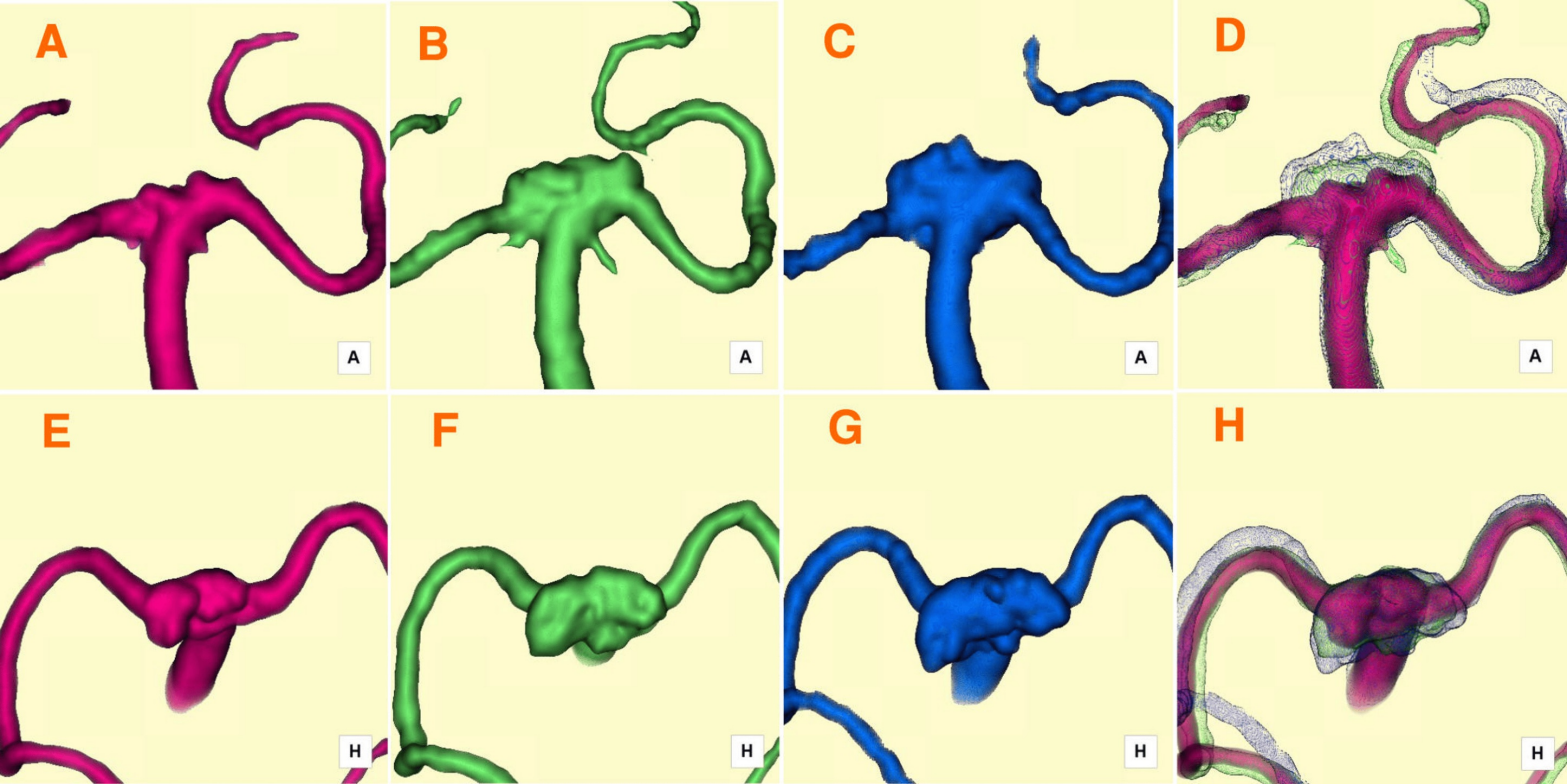
Serial 3D silent MRA images capturing the progression of RC enlargement A, E) Fourteen days post-treatment: The RC formed and extended superoposteriorly, as shown in anterior-posterior (A) and superior-inferior (E) projections. B, F) Six months post-treatment: The RC expanded postero-laterally (horizontally), as depicted in anterior-posterior (B) and superior-inferior (F) projections. C, G) One year post-treatment: The RC exhibited continued postero-lateral (horizontal) growth, along with an additional minor superior (vertical) extension at its central region, as observed in anterior-posterior (C) and superior-inferior (G) projections. D, H) Fused images overlaying time-series 3D silent MRA data provide a comprehensive visualization of the progressive RC enlargement, shown in anterior-posterior (D) and superior-inferior (H) projections. MRA: magnetic resonance angiography; RC: residual cavity

Fourteen days post-treatment, the RC was observed extending from the anterior neck region toward the superoposterior direction (Figures 2A, 2E). By six months, the RC had expanded horizontally in the postero-lateral direction (Figures 2B, 2F). After one year, further horizontal growth occurred, with minor vertical extension in the central RC (Figures 2C, 2G). Time-series 3D silent MRA fused images effectively visualized RC enlargement over time (Figures 2D, 2H). Hemodynamic analysis of the RC was performed using the CFD package Hemoscope v1.4 (EBM & AMIN Corp., Tokyo, Japan), following the established methodology [9]. Parameters such as RC volume (mm³), surface area (mm³), neck size (mm), depth (mm), and aspect ratio were evaluated. RC volumes increased from 42.15 mm³ at 14 days to 155.75 mm³ at six months and 293.45 mm³ at one year. Hemodynamic parameters included streamlines, pressure drop (mmHg), flow rate (m/s), wall shear stress magnitude (WSSm, Pa), and wall shear stress vector direction (WSSv, degrees).

**Figure 3:**
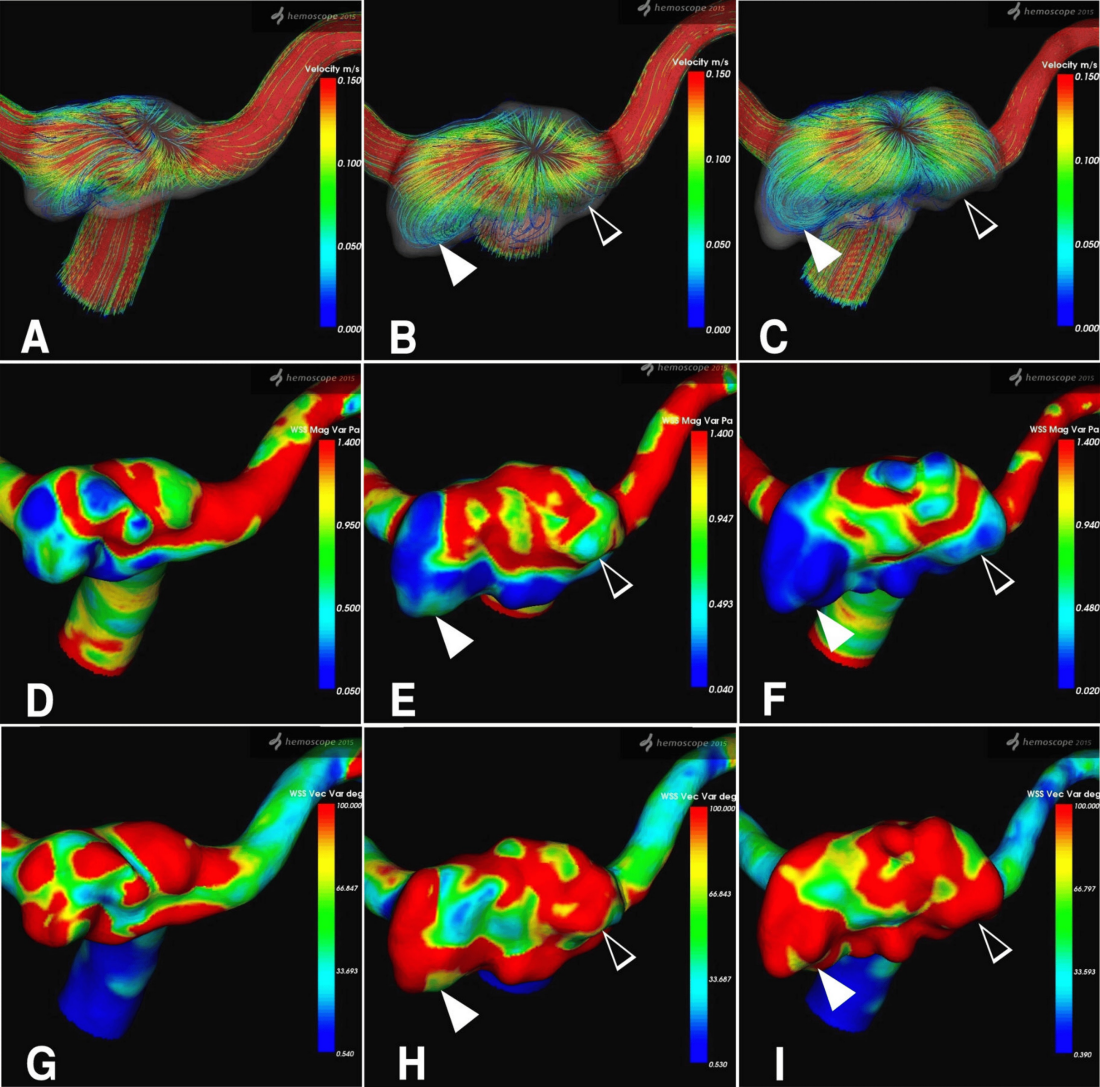
Hemodynamic assessment of the RC, demonstrating temporal changes in the streamline, WSSm, and WSSv parameters A-C) Sequential images of streamlines viewed from the superior-inferior direction at 14 days, 6 months, and 1-year post-treatment, respectively. The color bar on the right indicates velocity (m/s), ranging from 0.150 (red, representing higher velocity) to 0.000 (blue, representing lower velocity). A high-velocity streamline (red) is observed centrally in the remnant, streaming in and out like a fountain. In contrast, reduced streamline velocity (blue) is seen in the left postero-lateral region (white arrowhead) and the right lateral region (empty white arrowhead) of the enlarged RC. D-F) Sequential images of WSSm at 14 days, 6 months, and 1 year post-treatment, respectively. WSSm (Pa) is represented by a color bar ranging from 1.400 (red) to 0.050 (blue). A decrease in WSSm is evident in the left postero-lateral region (white arrowhead) and the right lateral region (empty white arrowhead) of the enlarged RC. G-I) Sequential images of WSSv at 14 days, 6 months, and 1-year post-treatment, respectively. WSSv (degree) is displayed with a range of 100.000 (red) to 0.540 (blue). An increase in WSSv is shown in the left postero-lateral region (white arrowhead) and the right lateral region (empty white arrowhead) of the enlarged RC. RC: residual cavity; WSSm: wall shear stress magnitude; WSSv: wall shear stress vector

Temporal changes in RC dynamics were assessed over 14 days, 6 months, and 1 year. Fourteen days post-procedure, streamlines viewed from the superior-inferior direction revealed inflow from the neck into the RC, accompanied by primary outflow (Figure 3A). At six months, a high-velocity jet flow entered the RC at the neck center in a fountain-like pattern, while the RC expanded horizontally into a relatively flat shape (Figure 3B). After one year, these patterns persisted, with high-velocity streamlines at the neck center and low-velocity flow within the bilaterally and posteriorly extended RC (Figure 3C). Temporal variations in WSSm and WSSv mirrored streamline findings. The central RC near the neck exhibited relatively high WSSm and low WSSv, while the distal RC showed low WSSm and high WSSv.

**Figure 4:**
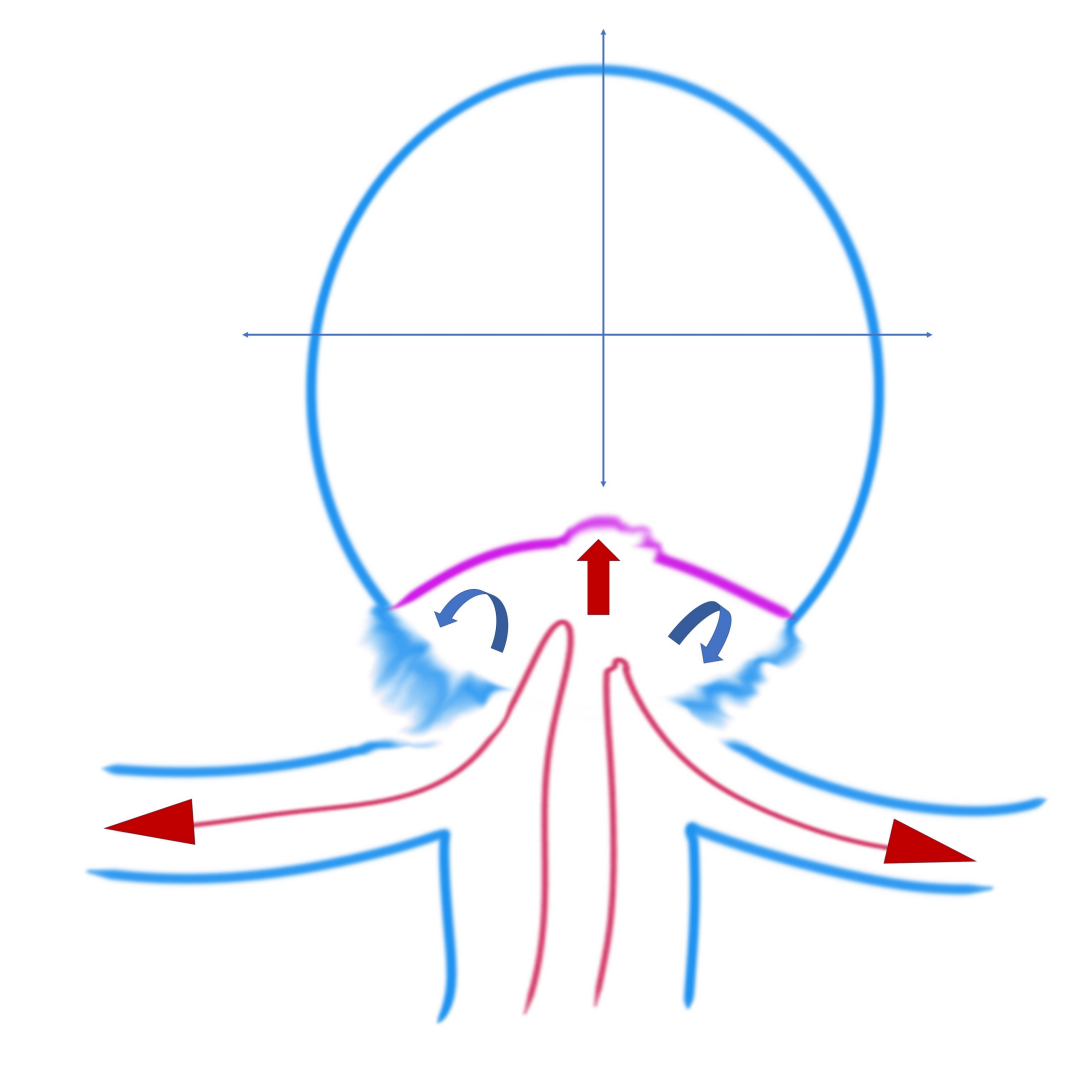
Figure 4: Illustration of the hemodynamic mechanism of RC enlargement The basilar artery blood flow (long red arrows) enters the RC, with the main blood flow (red arrow) within the RC impacting the newly formed intimal surface at the center of the RC. Note the detached blood flow (curved blue arrows) on both sides of the RC. RC: residual cavity Image Credits: Toru Satoh

The hemodynamic mechanism of RC enlargement due to detached secondary flow is schematically depicted in Figure 4.

## Discussion

In coil embolization of cerebral aneurysms, a neo-endothelial surface at the aneurysm neck, blockage of blood flow, and complete thrombus formation within the coiled aneurysm are considered critical for achieving durable occlusion [3,10]. However, due to the complex morphology of aneurysms, complete filling of the aneurysm sac is often challenging, leading to the formation of RCs at the neck after coiling [1]. Even when initial occlusion appears successful, recurrence rates of 21% [11] to 33% [12] have been reported during long-term follow-up, with recurrence typically attributed to either coil compaction or aneurysm sac growth.

Coil compaction has been widely studied as a contributor to recurrence. Asai et al. reported that coils generally occupy about 30% of the aneurysm cavity, with the remaining 70% filled by thrombus, forming a stable coil-thrombus complex [4]. While experiments in silicone aneurysms suggested that coil shape remained stable under constant pressure, pressure fluctuations from blood pressure and pulsatile flow were shown to induce compaction. In contrast, Hoppe et al. emphasized that factors such as the degradation of unstable thrombus, persistent blood flow through the coil-thrombus complex, and incomplete neo-intima formation at the aneurysm neck may play a more significant role in aneurysm sac growth than coil compaction [5]. Clinical studies have reported mixed findings. Abdihalim et al. observed coil compaction in 11 out of 29 cases and sac growth in 18 cases [13]. Similarly, Wang et al. found coil compaction in four cases and sac growth in five cases based on a histopathological examination of nine recurrent aneurysms [6]. These findings suggest that sac growth may often dominate in cases of recurrence.

The formation and enlargement of RCs post-coiling are closely linked to the aneurysm’s morphology and hemodynamics, both pre and post-embolization [14]. Sugiyama et al. identified high inflow rates from the basilar artery and coil packing densities below 30% as significant factors for recurrence in basilar-tip aneurysms [15]. Luo et al. showed that high WSS and flow velocity at the aneurysm neck disrupted the internal elastic membrane and thinned the media, contributing to wall damage and preventing effective thrombus formation [16]. In such cases, persistent high-pressure and high-velocity flows cause coil compaction, resulting in RC formation. Ishii et al. further demonstrated that RC enlargement is often associated with high-pressure flow impingement on the coil core, particularly in aneurysms with large neck areas [17]. Suzuki et al., using silent MRA, reported that pressure distribution varied depending on RC location, with wall pressure localized on the aneurysm sac in sac growth cases and on the coil surface in RC enlargement cases [18].

In this case, the hemodynamic mechanisms contributing to RC formation and enlargement are likely multifactorial. Initially, coil compaction due to repeated flow impingement at the neck led to RC formation within 14 days post-procedure. Pulsatile flow and kinetic energy conversion to static pressure at the neo-endothelial surface induced vertical RC enlargement. From six months to one year post-treatment, RC enlargement shifted horizontally. Flow impinging at the center of the neck detached at the distal edge, creating secondary low-velocity flows, as illustrated in Figure 4. These secondary flows formed vortices that promoted erythrocyte stagnation and inflammatory cell infiltration, weakening the aneurysm wall and contributing to distal sac expansion [2,8]. To the best of our knowledge, no case has been reported suggesting, from a hemodynamic perspective, the involvement of secondary flow detached from the primary jet flow in the growth of RCs following coil embolization of cerebral aneurysms. In this case, rapid remnant enlargement was observed following coil-only treatment. Hemodynamic analysis suggested that in addition to coil compaction at the inflow area caused by the primary blood flow, the growth was likely influenced by a secondary flow. Since remnant growth does not follow a consistent pattern, retreatment strategies should consider both morphological and hemodynamic evaluations.

## Conclusions

In this study, we performed a time-course analysis of RC morphology in a basilar-tip aneurysm treated with coils, utilizing silent MRA and CFD to explore the mechanisms underlying RC formation and enlargement. This case highlights that the vertical expansion of the RC was likely driven by coil compaction due to repetitive flow impingement while its horizontal expansion appeared to result from flow detachment, leading to wall weakening and subsequent bilateral aneurysm sac expansion. These hemodynamic factors may explain the RC formation and enlargement observed post-coiling in this study. Incorporating CFD analysis with silent MRA offers significant insights into the hemodynamic factors influencing cerebral aneurysms after coil embolization.

## References

[1] Ferns SP, Sprengers ME, van Rooij WJ (2009). Coiling of intracranial aneurysms: a systematic review on initial occlusion and reopening and retreatment rates. Stroke.

[2] Jeong W, Rhee K (2012). Hemodynamics of cerebral aneurysms: computational analyses of aneurysm progress and treatment. Comput Math Methods Med.

[3] Brinjikji W, Kallmes DF, Kadirvel R (2015). Mechanisms of healing in coiled intracranial aneurysms: a review of the literature. AJNR Am J Neuroradiol.

[4] Asai T, Nagano Y, Ohshima T, Miyachi S (2022). Experimental study of coil compaction: impact of pulsatile stress. J Neuroendovasc Ther.

[5] Hoppe AL, Raghavan ML, Hasan DM (2015). Comparison of the association of sac growth and coil compaction with recurrence in coil embolized cerebral aneurysms. PLoS One.

[6] Wang C, Li M, Chen H, Yang X, Zhang Y, Zhang D (2022). Histopathological analysis of in vivo specimens of recurrent aneurysms after coil embolization. J Neurointerv Surg.

[7] Satoh T, Hishikawa T, Hiramatsu M, Sugiu K, Date I (2019). Visualization of aneurysmal neck and dome after coiling with 3D multifusion imaging of silent MRA and FSE-MR cisternography. AJNR Am J Neuroradiol.

[8] Satoh T, Sugiu K, Hiramatsu M, Haruma J, Date I (2024). Evaluation of the shrinkage process of a neck remnant after stent-coil treatment of a cerebral aneurysm using silent magnetic resonance angiography and computational fluid dynamics analysis: illustrative case. J Neurosurg Case Lessons.

[9] Satoh T, Yagi T, Sawada Y, Sugiu K, Sato Y, Date I (2022). Association of bleb formation with peri-aneurysmal contact in unruptured intracranial aneurysms. Sci Rep.

[10] Grüter BE, Wanderer S, Strange F (2021). Patterns of neointima formation after coil or stent treatment in a rat saccular sidewall aneurysm model. Stroke.

[11] Raymond J, Guilbert F, Weill A (2003). Long-term angiographic recurrences after selective endovascular treatment of aneurysms with detachable coils. Stroke.

[12] Murayama Y, Nien YL, Duckwiler G (2003). Guglielmi detachable coil embolization of cerebral aneurysms: 11 years' experience. J Neurosurg.

[13] Abdihalim M, Watanabe M, Chaudhry SA, Jagadeesan B, Suri MF, Qureshi AI (2014). Are coil compaction and aneurysmal growth two distinct etiologies leading to recurrence following endovascular treatment of intracranial aneurysm?. J Neuroimaging.

[14] Damiano RJ, Tutino VM, Paliwal N (2020). Aneurysm characteristics, coil packing, and post-coiling hemodynamics affect long-term treatment outcome. J Neurointerv Surg.

[15] Sugiyama S, Niizuma K, Sato K (2016). Blood flow into basilar tip aneurysms. A predictor for recanalization after coil embolization. Stroke.

[16] Luo B, Yang X, Wang S (2011). High shear stress and flow velocity in partially occluded aneurysms prone to recanalization. Stroke.

[17] Ishii T, Fujimura S, Takao H (2021). Hemodynamic and morphologic factors related to coil compaction in basilar artery tip aneurysms. World Neurosurg.

[18] Suzuki T, Genkai N, Nomura T, Abe H (2020). Assessing the hemodynamics in residual cavities of intracranial aneurysm after coil embolization with combined computational flow dynamics and silent magnetic resonance angiography. J Stroke Cerebrovasc Dis.

